# Study protocol for investigating real-world implementation of a combined glial fibrillary acidic protein (GFAP) and ubiquitin carboxy-terminal hydrolase L1 (UCH-L1) blood test in the management of adult mild traumatic brain injury in a single-centre European emergency department: the IMPACTS-BRAINI study

**DOI:** 10.1136/bmjopen-2026-120021

**Published:** 2026-06-18

**Authors:** Alfonso Lagares, Monica Maldonado, Andreea Baciu, Leandro Tosi, Carlos Loynaz, Sofia Martinez, Ana María Castaño León, Julia Hernández-Sánchez, Noelia García Barrio, Javier de la Cruz, Sofia Sánchez, Ana Lopez Jiménez, Cecilia Cueto-Felgueroso, Laura Carrasco, Eva Marquez, Alba María Fernández del Pozo, Cecilia Amil, Alejandro Arias, Odile Mejan

**Affiliations:** 1 Department of Neurosurgery, Hospital Universitario 12 de Octubre, Madrid, Community of Madrid, Spain; 2 Instituto de Investigación Sanitaria imas12, Hospital Universitario 12 de Octubre, Madrid, Spain; 3 Department of Surgery, Facultad de Medicina, Universidad Complutense de Madrid, Madrid, Spain; 4 Digital Health Group, Instituto de investigación Sanitaria imas12, Hospital Universitario 12 de Octubre, Madrid, Comunidad de Madrid, Spain; 5 Servicio de Análisis Clínicos-Bioquímica Clínica, Hospital Universitario 12 de Octubre, Madrid, Community of Madrid, Spain; 6 R&D Immunoassays, Chemin de l'Orme, bioMérieux, Marcy l'Etoile, France

**Keywords:** ACCIDENT & EMERGENCY MEDICINE, Brain Injuries, Clinical Protocols, Emergency Service, Hospital

## Abstract

**Introduction:**

Glial fibrillar acidic protein (GFAP) and ubiquitin carboxy-terminal hydrolase L1 (UCH-L1) have been shown to rule out CT-detectable intracranial lesions in patients with mild traumatic brain injury (mTBI) when assessed within the first 12 hours after injury. These biomarkers have been validated across different laboratory-based and point-of-care testing platforms. The combined biomarker test has been incorporated into several mTBI management algorithms in Europe. However, data regarding its real-world application, including patients with neurological comorbidities and its impact on reducing CT utilisation or emergency department (ED) length of stay, are lacking. This study will evaluate the performance of an automated laboratory-based assay for serum GFAP and UCH-L1 when integrated into a standardised clinical pathway for the diagnostic management of patients with mTBI. In addition, the study will assess its potential value in reducing CT scan prescription and ED time.

**Methods and analysis:**

This single-centre observational study, with prospective data collection before and after the implementation of a combined test measuring GFAP and UCH-L1 in the clinical laboratory, will be conducted at Hospital Universitario 12 de Octubre, Madrid, Spain. Patients with clinically defined mTBI will be managed according to a newly implemented clinical pathway including biomarker testing. mTBI will be defined using predefined clinical criteria including a plausible traumatic mechanism, Glasgow Coma Scale score 13–15 assessed 30 min or more after injury, and compatible signs and/or symptoms of brain injury. Eligible patients must undergo blood sampling within 12 hours of injury and before imaging prescription. The effectiveness of this management approach will be compared with a previously established cohort of patients, prospectively enrolled under identical inclusion and exclusion criteria. A cohort of 1000 patients with mTBI, in whom biomarker testing was used in their management, will be included in the post implementation group. The pre-implementation cohort will be drawn from a comparable time period. The primary outcome measures are: the diagnostic performance of GFAP and UCH-L1, measured using an automated assay, for discriminating between patients with positive and negative findings on brain CT scans; the safety of the new clinical pathway in terms of complications such as unexpected surgery or deterioration, as well as the reduction in CT use after the implementation. Secondary objectives will be reduction in ED times and direct costs, as well as physicians’ compliance with the algorithms.

**Ethics and dissemination:**

The study was reviewed by the Institutional Research Committee of Hospital 12 de Octubre, Madrid, Spain (Ref TP25/0144) and deemed exempt from formal ethical approval and informed consent requirements. This study’s results will be presented at national and international meetings, including meetings of patient associations, and published in peer-reviewed journals.

**Trial registration number:**

NCT07311486.

**STRENGTHS AND LIMITATIONS OF THIS STUDY**

- Prospective single-centre emergency department (ED) study embedding an automated laboratory-based combined glial fibrillar acidic protein and ubiquitin carboxy-terminal hydrolase L1 assay into a predefined clinical pathway for adult mild traumatic brain injury (mTBI) management.
- The absence of exclusion criteria based on comorbidities enhances the generalisability of the results and supports the applicability of the clinical pathway across a broad adult mTBI population.
- The pragmatic study design allows assessment of whether implementation of the biomarker-guided pathway is associated with changes in CT utilisation and ED length of stay while maintaining patient safety within routine clinical practice.
- Variability in radiologists’ interpretation of head CT scans may influence outcome classification and could affect estimates of the diagnostic performance of the combined biomarker test.
- Variability in physicians’ adherence to the clinical algorithm may reduce the ability to detect differences between the standard and biomarker-integrated pathways.

## Introduction

 Mild traumatic brain injury (mTBI) is one of the main causes of consultation to the emergency departments (EDs), affecting millions of patients every year worldwide.^1^ The management of mTBI is based on the need for CT prescription.^2^,^4^ Physicians must assess each patient’s risk of harbouring CT-detectable intracranial lesions that could lead to neurological deterioration.^5^,^7^ CT is prescribed if specific conditions are met by the patient. The use of these Clinical Decision Rules (CDRs) is not always easy, as they depend on the assessment of symptoms and conditions that are sometimes difficult to determine.^2^ Therefore, there is a wide variability in using the different CDRs among physicians.^2 3^ In addition, fear of missing a brain injury or pressure from patients or relatives may lead physicians to disregard CDRs and use CT more broadly. Consequently, there is a clear overuse of CT in the ED in the screening of mTBI.^8 9^ In addition, patients receiving antithrombotic and antiplatelet treatments undergo CT scans and are hospitalised more often after mTBI.^2 10^

As part of an effort to enhance CDR with more objective tools, blood biomarkers have been proposed as a possible objective parameter that could be assessed easily in a short time and be reliable to rule out the presence of CT-detectable intracranial lesions and therefore the need for a CT scan in these patients.^11 12^ Blood biomarkers embedded in CDRs or as part of clinical guidelines may increase the objectivity of assessment and support the safe management of mTBI in the ED. Serum S-100B has long been included in the Scandinavian Guidelines for the initial management of mTBI, and its utility in assessing patients at intermediate risk has been demonstrated.^13 14^ However, the use of S100B has not gained generalised acceptance due to its decreasing sensitivity over time, which limits its use to a very narrow window (within 3 hours of the injury), as well as decreased specificity in patients affected by systemic trauma.^15^ A test combining the determination of two brain-specific proteins, ubiquitin carboxy-terminal hydrolase L1 (UCH-L1) and glial fibrillar acidic protein (GFAP), has been designed and validated for predicting the absence of lesions on CT scans in patients, ruling out the need for CT scanning and therefore theoretically reducing unnecessary CT scanning.^15 16^ Prior studies suggest that, using validated biomarker thresholds, patients can be safely managed in the ED, as the combined test demonstrates a negative predictive value approaching 100%, with a very low rate of false-negative results for ruling out CT-detectable intracranial lesions.^15^,^18^

Different automated platforms have been validated to date with the aim of bringing these new screening tools into clinical practice. These platforms vary in both the biological matrix analysed (plasma vs serum) and their potential implementation in the ED, either as point-of-care testing^19^,^21^ or via core laboratory analysis.^15 16 22^ Each platform has been supported by its own validation study, in which diagnostic performance has consistently been good. However, patients with neurological comorbidities have routinely been excluded from these studies, particularly individuals with cognitive impairment or pre-existing neurological or psychiatric disorders.^23^ At the same time, older adults are increasingly becoming the predominant population affected by mTBI, reflecting global population ageing and increased life expectancy.^124^,^26^ Neurological comorbidities are substantially more prevalent in this age group, raising important concerns about the generalisability of existing validation data to real-world clinical practice.^27^,^29^ Theoretical models suggest that incorporating the combined test into a clinical pathway for mTBI management in the ED could reduce length of stay and overall healthcare costs.^30^,^32^ However, to date, no real-world clinical data are available to substantiate these potential benefits.

Therefore, although the combined test has been extensively validated across different analytical platforms under controlled conditions, there is still no clinical study that robustly evaluates its real-world use in the management of mTBI—particularly with respect to its feasibility and applicability in routine practice, its diagnostic performance in all possible patients, and its association with clinically meaningful outcomes including CT utilisation and mTBI management timelines. This study aims to assess the feasibility, applicability and clinical performance of a structured clinical pathway for patients with mTBI presenting to the ED, incorporating the use of an in vitro diagnostic assay measuring serum GFAP and UCH-L1 within the first 12 hours postinjury to support clinical decision-making regarding head CT use. The primary objectives are: to evaluate the diagnostic performance of the VIDAS TBI test for ruling out CT-detectable intracranial lesions on head CT after mTBI; to assess the safety of the clinical pathway in terms of complications or unexpected neurological deterioration and to estimate changes in CT utilisation associated with implementation of the clinical pathway that includes biomarker testing. The secondary objectives are: to characterise time intervals in mTBI management and to compare management timelines before and after implementation of the clinical pathway, to estimate the direct costs associated with mTBI care at our centre and the economic implications associated with the new clinical pathway, and to evaluate physician adherence with the proposed diagnostic algorithm.

## Methods and analysis

### Study design

**Figure 1:**
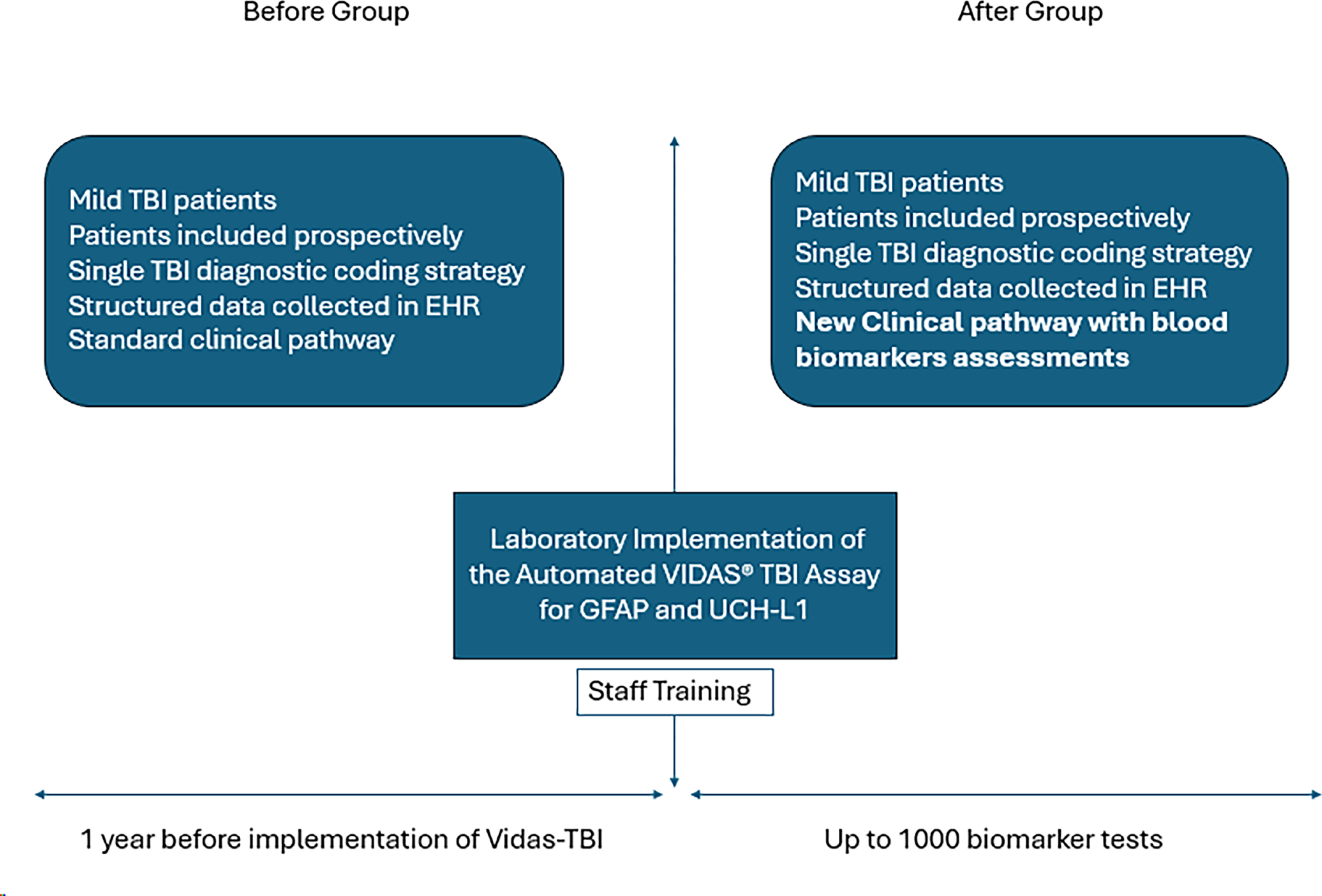
General scheme of the before–after study design. Patients with mTBI were managed according to a standard clinical pathway during the pre-implementation period and according to an updated pathway incorporating blood biomarker assessment during the post implementation period. The two periods were separated by the laboratory implementation of the VIDAS-TBI automated GFAP and UCH-L1 assay and associated staff training. EHR, electronic health record; GFAP, glial fibrillar acidic protein; mTBI, mild traumatic brain injury; UCH-L1, ubiquitin carboxy-terminal hydrolase L1.

The IMPACTS-BRAINI study is a single-centre observational study with prospective data collection conducted before and after the implementation of the VIDAS TBI (GFAP, UCH-L1) test in the clinical laboratory of Hospital Universitario 12 de Octubre(figure 1). The introduction of this assay enables the adoption of a new clinical pathway that incorporates combined GFAP and UCH-L1 biomarker testing into the management of patients with clinically defined mTBI. Accordingly, the study follows a before-and-after observational design with prospective data collection.

### Study setting

The IMPACTS-BRAINI study is part of the BRAINI2 project, co-funded by the European Union through the European Institute of Innovation and Technology (Health). This study is an investigator-initiated project conducted in collaboration with bioMérieux, which provided funding for the installation of a VIDAS TBI analyser and the associated biomarker tests. This prospective single-centre study will be conducted in the ED of Hospital 12 de Octubre, Madrid, Spain. Participant recruitment began in July 2024 with prospective enrolment and data collection during the pre-implementation phase. The VIDAS TBI (GFAP, UCH-L1) assay was implemented in July 2025, after which prospective enrolment and data collection continued during the post implementation phase. Completion of the post implementation cohort is anticipated in July 2026.

### Study population

Patients will be included in the study during two distinct time periods: before and after the implementation of a new clinical pathway including biomarker testing for the management of clinically defined mTBI. The pre-implementation phase is planned to last 1 year prior to introducing the new pathway. Patients will be included if they meet the following criteria: age ≥18 years, assessed for mTBI with a Glasgow Coma Score (GCS) of 13–15, in whom blood sampling could be obtained ≤12 hours after injury and before any imaging prescription. mTBI will be defined by at least criterion (a) and (d) of the following and criteria (b) or (c). (a) The presence of a plausible mechanism observed/or related by the patient’s recount of the injury event. (b) Presence of one or more clinical signs attributable to brain injury: loss of consciousness immediately following injury, alteration of mental status immediately following the injury, posttraumatic amnesia or any neurological abnormality. (c) At least two acute symptoms related to the injury: feeling confused or disoriented, headache, nausea, vomiting, dizziness, vision problems, memory problems, emotional lability or irritability. (d) GCS between 15 and 13, assessed 30 min or more after injury.

Patients will be excluded in the study if they have at least one of the following criteria: GCS score of 3–12 on admission, unknown time of injury, time since injury exceeding 12 hours, primary admission for a non-traumatic neurological disorder (such as stroke or spontaneous intracranial hematoma), penetrating head injury, brain tumour, mechanical ventilation from the trauma scene or prehospital management, venipuncture not feasible.

### Intervention

This study uses a before-and-after observational design to evaluate the implementation of a biomarker-guided clinical pathway for the management of clinically defined mTBI in the ED. In both the pre-implementation and post implementation periods, patients will meet the same clinically defined mTBI inclusion and exclusion criteria described in the Study population section. During the pre-implementation period, patients were managed according to standard care following national recommendations,^33^ which included clinician-guided decisions regarding head CT imaging without biomarker testing. Briefly, patients with pathological findings in neurological examination (GCS <15; focal neurological deficits, seizures) and risk factors included in different CDRs were prescribed a CT scan. These risk factors include loss of consciousness, post-traumatic amnesia, advanced age (≥65 years), antithrombotic treatments including platelet inhibitors, difficulties in assessing level of consciousness (due to cognitive impairment or intoxication), persistent symptomatology (such as repeated vomiting or persistent headache), high energy mechanisms (fall from over 1 m, car accident, pedestrian involved in traffic accident or accidents involving other seriously injured patients) and signs or symptoms of cranial base fracture or depressed skull fracture.

**Figure 2:**
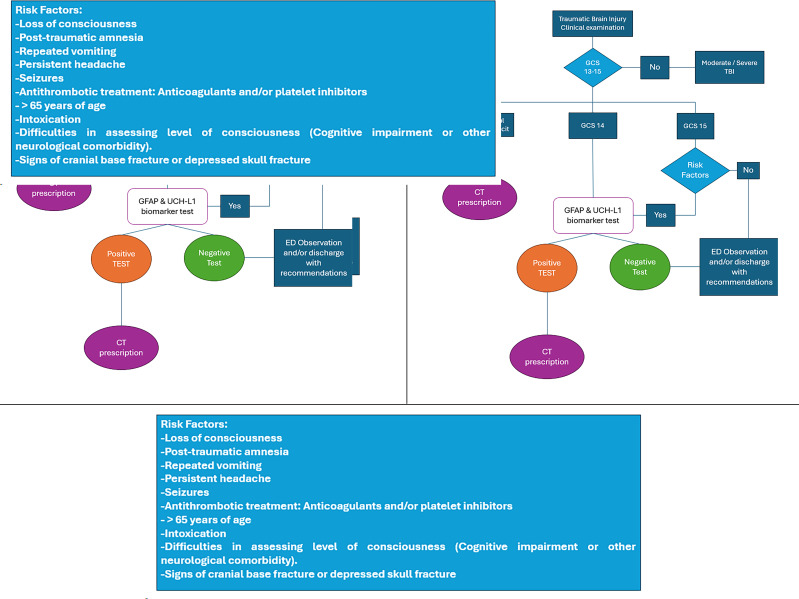
Standard clinical pathway (left) and updated pathway incorporating blood biomarker testing (right) for the management of mTBI. The updated pathway integrates GFAP and UCH-L1 testing into existing clinical decision-making. ED, emergency department; GFAP, glial fibrillar acidic protein; mTBI, mild traumatic brain injury; UCH-L1, ubiquitin carboxy-terminal hydrolase L1; GCS, Glasgow Coma Score.

Following laboratory implementation of the VIDAS TBI assay, a revised clinical pathway incorporating serum GFAP and UCH-L1 testing was introduced as part of routine clinical care (figure 2). Patients with GCS=13 assessed ≥30 min after injury are not managed using biomarker-guided CT decision-making because of the high observed prevalence of CT-detectable intracranial lesions in this subgroup in our previous and ongoing institutional experience, together with the expected turnaround time for biomarker results.

Before beginning the implementation phase, attending physicians received specific training on the new pathway. This training will be repeated every 3 months during the post implementation phase. No individual patient-level randomisation or alteration of usual clinical decision-making authority occurred; the pathway was implemented at the service level, and patient management remained at the discretion of the treating clinician. Therefore, clinicians were allowed to make independent decisions, including the option not to use biomarkers and ordering a CT scan directly if they deemed it necessary, or discharging patients with minimal trauma without requesting biomarker analysis or prescribing a CT scan.

### Study outcomes

The primary objectives of the study are: (1) to evaluate the performance of the combined GFAP and UCH-L1 biomarker assay in ruling out the need for head CT scanning after mTBI; (2) to assess the safety of the new biomarker-guided clinical pathway and (3) to determine the reduction in CT scan utilisation associated with implementation of this pathway. The corresponding primary outcome measures are: (1) diagnostic performance of the biomarker assay, expressed in terms of sensitivity, specificity, positive and negative predictive values, likelihood ratios, and the lower limits of the 95% CIs, using head CT findings (positive vs negative for CT-detectable intracranial lesions) as the reference standard; (2) occurrence of neurological deterioration within 2 weeks following mTBI, defined as unexpected neurological worsening or an unplanned need for hospital admission or neurosurgical intervention and (3) the absolute number and proportion of patients undergoing head CT during the two study periods (before and after implementation of the new clinical pathway).

Secondary outcomes include differences in time spent on the management of mTBI in the ED, differences in direct healthcare costs and physician compliance with the biomarker-guided clinical pathway. Time intervals related to mTBI management will be obtained directly from the hospital’s information systems. Direct cost consumption will be extracted for each ED episode among included patients and compared between the pre-implementation and post implementation periods of the clinical pathway.

Compliance with the protocol will be defined as the proportion of cases in which head CT ordering and/or biomarker prescription aligns with the pathway recommendation. Compliance rates will be further stratified according to the results of the combined GFAP and UCH-L1 biomarker test.

### Data collection and monitoring

Data obtained as standard of care for the management of mTBI will be collected in the electronic clinical records. The Electronic Health Record (EHR) is an essential tool for clinical decision-making and data collection, as it enables accurate and systematic documentation of patient information. However, its full potential is exploitable and useful when the data are structured. For this reason, and since the TBI information will be collected in the EHR, the hospital’s information technology data area improved the structuring and standardisation of the information in the EHR. To this end, the mTBI diagnostic code was unified and codified (SNOMED CT), and a structured form was created to capture the questions typically asked when attending to a patient with mTBI. The completeness of the defined diagnosis, together with the designed form, will allow the identification of the cohort of patients with mTBI. Data collection included in the structured form for prospective analysis comprises demographic and baseline information, including presence or absence of comorbidities, concomitant medications (particularly antithrombotic medications), cause and mechanism of the injury, clinical symptoms and signs of mTBI, and the presence of associated systemic trauma. In addition to the data captured in the form, the study will also collect the reason to prescribe a brain CT scan and immediate CT findings by the local radiologist. CT will be classified as positive if there is a presence of haemorrhagic traumatic lesions or depressed skull fracture in the CT, negative or not performed. All analytical data, including biomarker concentrations and results of the combined test result, are automatically recorded in the EHR and included in the study dataset. At 2 weeks after TBI, clinical research associates will review all available information regarding patients’ neurological status, as well as any need for hospitalisation or subsequent ED visits related to mTBI. Follow-up data will be obtained through automatic extraction from the institutional electronic health record and review of regional healthcare records available through the Madrid health information systems, including the HORUS platform, which provides access to hospital and primary care records. Regular quality checks on the information consigned in the EHR by attending physicians will be performed as a programme for quality improvement of the mTBI bundle, at least every 3 months. This programme has already been implemented by the Department of Neurosurgery to enhance the collection of structured data in clinical records at our hospital. Data collection regarding mTBI patients managed in our hospital began at least 1 year before the adoption of the mTBI clinical pathway, in order to establish the pre-implementation patient group.

The deidentified data from the mTBI cohort will be obtained and transferred into a web-based electronic CRF built on REDCap for analysis. The REDCap database will contain pseudo-anonymised clinical information on patients with mTBI from 1 year prior to the implementation of the protocol and from the post implementation period.

### Sample size

The IMPACTS-BRAINI study is a pragmatic, single-centre, before/after implementation study; accordingly, the sample size is primarily defined by the number of consecutively eligible mTBI patients presenting during the predefined pre-implementation and post implementation periods. Sample size considerations also account for the precision of diagnostic performance estimates for a rule-out strategy. Assuming an intracranial lesion prevalence of approximately 10% on head CT, inclusion of about 1000 patients would be expected to yield roughly 100 CT-positive cases, enabling clinically meaningful precision around sensitivity and negative predictive value estimates. In addition, with a baseline CT utilisation rate of 70%, this sample size would allow detection of clinically meaningful reductions in CT use in the post implementation period, particularly for absolute reductions of approximately 15% or greater. For the implementation (before–after) evaluation, we anticipated approximately 2000 patients in the pre-implementation period and 1000 patients managed using the biomarker-guided pathway post implementation. Assuming a two-sided α of 0.05 and 80% power, this sample size of 1000 patients managed using a biomarker-embedded algorithm that provides the ability to detect a difference in ED length of stay of approximately 0.11 SD between periods, corresponding to roughly 20–35 min for typical variability in ED length of stay.

### Statistical considerations

The predictive performance of the predefined cut-off values for the two biomarkers will be validated in the entire study population, calculating sensitivity, specificity, negative predictive value, positive predictive value and diagnostic accuracy for CT-detectable intracranial lesions. Receiver operating characteristic (ROC) curve analyses will also be performed, and the area under the ROC curve of GFAP, UCH-L1 and the combined test will be estimated as complementary measures of discriminatory performance. Patients in whom no head CT is performed will be classified as CT-negative. In addition, the clinical effectiveness of the biomarker-guided strategy will be evaluated by comparing CT utilisation rates between the pre-implementation and post implementation cohorts. Safety of the test will be evaluated through the occurrence of neurological deterioration, hospitalisation or subsequent ED visits within 2 weeks following mTBI.

The influence of age on diagnostic performance will be examined by stratifying patients into age groups (<65 and ≥65 years), with further subgroup analyses among patients aged ≥65 years (65–79 years and ≥80 years). Neurological comorbidities, including cognitive impairment, previous stroke, neurodegenerative disease, epilepsy, and chronic neurologic or psychiatric disorders documented in the medical record, will be identified. Subgroup and exploratory analyses will compare biomarker concentrations and diagnostic performance metrics of the predefined cutoffs between patients with and without neurological comorbidities.

To account for potential imbalances between the pre-implementation and post implementation groups with respect to demographic characteristics, comorbidities, treatments or mTBI-related signs and symptoms, multivariable analyses will be performed as needed to adjust for confounding and to ensure comparability between groups. Secondary outcomes will likewise be analysed with stratification by age and comorbidity status.

The study will be reported in accordance with the Strengthening the Reporting of Observational Studies in Epidemiology (STROBE), Consolidated Standards of Reporting Trials (CONSORT) and Standard Protocol Items: Recommendations for Interventional Trials (SPIRIT) guidelines.

### Patients and public community involvement

Patient associations are partners of the BRAINI2 consortium and are part of the study’s governing structure (General Assembly). They are involved in this research's design and planning.

## Ethics and dissemination

This study evaluates the implementation of a biomarker-guided diagnostic pathway within routine clinical care for patients with mTBI. All diagnostic and management decisions followed standard clinical practice, and no additional procedures or deviations from usual care were required for study purposes. Data are collected prospectively from routine clinical records and fully deidentified prior to analysis. Given the observational nature of the study, the use of standard-of-care management and the analysis of deidentified data, the study was reviewed and approved by the institutional research committee Hospital 12 de Octubre, Madrid, Spain on 26 March 2025 (Ref TP25/0144) and determined to be exempt from formal ethical approval and from the requirement for informed consent. This study’s results will be presented at national and international meetings, including meetings of patient associations, and published in peer-reviewed journals.

## Discussion

mTBI represents a significant burden on hospitals and EDs in developed countries.^1 34^ There is a clear need to improve the management of these patients, as physicians often face uncertainty regarding the safety of discharge home, variability in clinical decision-making^2 3 10^ and inefficient use of resources such as CT imaging and ED time.^8 9 35^ An objective measure capable of supporting physicians in managing mTBI in a safe, efficient and rigorous manner could substantially improve care delivery.^36^

The study will evaluate the real-world clinical implementation and use of biomarker testing in patients with mTBI. Findings from several large clinical studies already published—Evaluation of Biomarkers of Traumatic Brain Injury (ALERT-TBI), Collaborative European NeuroTrauma Effectiveness Research in Traumatic Brain Injury (CENTER-TBI), Transforming Research and Clinical Knowledge in Traumatic Brain Injury (TRACK-TBI) and Blood biomarkers to improve the management of mild traumatic BRAIN Injury (BRAINI)—will contribute to the interpretation and discussion of this real-world validation study.^1516 37^,^43^ Collectively, these studies provide evidence supporting the value of the biomarkers GFAP and UCH-L1 for ruling out the presence of intracranial injury following mTBI, despite differences in biomarker sampling times, measurement methodologies and patient age distributions.

Age is a particularly critical factor, as test specificity decreases with increasing age, a key finding highlighted by the BRAINI study.^15^ This study demonstrated the need to adjust cut-off values for the combined biomarker test in older adults. The forthcoming publication of the BRAINI-2 results, which validate new cut-offs for patients over 65 years of age, will be incorporated into the discussion of the present study’s findings.^23^

The current study aims to determine the real-world clinical value of a laboratory-based test measuring both GFAP and UCH-L1 biomarkers for improving patient management in the ED. Laboratory-based testing may involve longer sample processing and turnaround times, and this limitation should be addressed in comparison with alternative testing locations or strategies, in light of the study results.

This study has several limitations. There will be no central adjudication of CT scan readings, which may increase variability in CT interpretation and potentially affect the diagnostic performance of the biomarker test. However, a pragmatic approach was intentionally adopted, as real-world clinical decisions rely on the judgement of the attending radiologist. Additionally, variability among physicians in adopting the new clinical pathway may influence biomarker utilisation and CT ordering practices. Educational sessions will be conducted during the implementation phase to promote adherence to the pathway and reduce physician-related variability. Compliance with the new pathway will be evaluated, and sensitivity analyses will be performed to estimate the potential impact of non-compliance on study outcomes.
