# Supplementary material for: Study protocol for investigating real-world implementation of a combined glial fibrillary acidic protein (GFAP) and ubiquitin carboxy-terminal hydrolase L1 (UCH-L1) blood test in the management of adult mild traumatic brain injury in a single-centre European emergency department: the IMPACTS-BRAINI study
Source: BMJ Open. 2026 Jun 18;16(6):e120021. doi: 10.1136/bmjopen-2026-120021 (PMC13289081; doi:10.1136/bmjopen-2026-120021)
Supplement: Supplementary file 1 [file bmjopen-2026-120021.reviewer_comments.pdf]

PEER REVIEW HISTORY

BMJ Open publishes all reviews undertaken for accepted manuscripts. Reviewers are asked to complete a checklist review form and are provided with free text boxes to elaborate on their assessment. These free text comments are reproduced below.

ARTICLE DETAILS

Title (Provisional)

Study protocol for investigating real-world implementation of a combined glial fibrillary acidic protein (GFAP) and ubiquitin carboxy-terminal hydrolase L1 (UCH-L1) Blood Test in the management of adult Mild Traumatic Brain Injury in the Emergency Department: the IMPACTS-BRAINI study

Authors

Lagares, Alfonso; Maldonado, Monica; Baciu, Andreea; Tossi, Leandro; Loynaz, Carlos; Martinez, Sofia; Castaño León, Ana María; Hernández-Sánchez, Julia; García-Barrio, Noelia; de la Cruz, Javier; Sánchez, Sofia; Lopez Jiménez, Ana; Cueto-Felgueroso, Cecilia; Carrasco, Laura; Marquez, Eva; María Fernández del Pozo, Alba; Amil, Cecilia; Arias, Alejandro; Mejan, Odile

---

VERSION 1 - REVIEW

**Reviewer**	**1**
**Name**	**Maiga, Amelia**
**Affiliation**	**Vanderbilt University Medical Center**
**Date**	**03-May-2026**
**COI**	**None**

This paper describes a planned (not completed) observational study looking at the implementation of biomarker aid-in-diagnosis study including patients with neurological comorbidities. I have the following comments:

1. In the Abstract, state clearly whether the “mTBI” population you plan to enroll constitute suspected mTBI or confirmed mTBI — and if the latter, how are you confirming this mTBI diagnosis prior to their prospective enrollment in the study?

2. Similarly, please better clarify whether your usage in the Methods section of the “mTBI” inclusion population = mild TBI, or suspected mild TBI. These are different clinical entities.

3. Please be more careful about using causal language in this non-randomized, observational study design (e.g., “its impact on clinically meaningful outcomes”). There are many other such examples.

4. How do the authors propose to obtain a GCS from all patients “30 minutes after injury”? Can you provide preliminary data demonstrating that it is feasible to obtain a GCS that close to the time of injury? Are these pre-hospital GCS values?

5. In the first paragraph of the “Intervention” section, it is not clear whether the pre-intervention study population overlaps with the mTBI definition provided in the “Study population” section. It appears to be different, which is a critical study design weakness.

6. The authors use of past/present/future tenses in the Methods section raises confusion about which parts of the study have already been accomplished. Are you actively collecting data in the “post” time period? Was the “pre” phase actually done prospectively, or do the authors intend to just extract data from a historical population in their database?

7. In general, the Methods section does not provide sufficient detail to meet the standards for reproducible research. For example: “The effect of neurological comorbidities on test performance will also be assessed”. There are many others.

8. How do the authors plan to capture “occurrence of neurological deterioration within two weeks following mTBI”? Will all enrolled patients, pre- and post-intervention, be actively contacted to determine whether or not one of these events has occurred? There is an EHR-check mentioned at “10-day post-TBI”, but this does not align with the secondary safety outcome described earlier in the Methods section.

9. It appears from Figure 2 that the authors only plan to do the biomarker test on GCS 14 patients and some GCS 15 patients, but not on GCS 13 patients. Please better explain this decision-making, particularly in light of the robust evidence for using biomarkers to rule out the need for head CTs in suspected TBI patients with GCS 13-15.

Minor

1. As a small point of clarification, GFAP/UCH-L1 rule out *CT-positive* intracranial lesions. They do not rule out MRI-positive intracranial lesions. This is important to state clearly in your Abstract and throughout.

2. Please define CDR at its first usage

VERSION 1 - AUTHOR RESPONSE

Reviewer: 1
Dr. Amelia Maiga, Vanderbilt University Medical Center
Comments to the Author:
This paper describes a planned (not completed) observational study looking at the implementation of biomarker aid-in-diagnosis study including patients with neurological comorbidities. I have the following comments:

1. In the Abstract, state clearly whether the “mTBI” population you plan to enroll constitute suspected mTBI or confirmed mTBI — and if the latter, how are you

confirming this mTBI diagnosis prior to their prospective enrollment in the study?

Response: Thanks for this comment, which required clarification. We plan to recruit patients with *clinically defined* mTBI rather than suspected mTBI, as eligibility requires fulfilment of predefined diagnostic criteria specified in the Methods section. These criteria include a plausible traumatic mechanism, compatible clinical signs and/or symptoms attributable to brain injury, and a Glasgow Coma Scale (GCS) score of 13–15 assessed ≥30 minutes after injury. To clarify this point, the Abstract has been revised as follows:

Page 6 ¶ 2: "This single-centre observational study, with prospective data collection before and after the implementation of a combined test measuring GFAP and UCH-L1 in the clinical laboratory, will be conducted at Hospital Universitario 12 de Octubre, Madrid. Patients with clinically defined mTBI will be managed according to a newly implemented clinical pathway including biomarker testing. mTBI will be defined using predefined clinical criteria including a plausible traumatic mechanism, Glasgow Coma Scale score 13–15 assessed 30 minutes or more after injury, and compatible signs and/or symptoms of brain injury. Eligible patients must undergo blood sampling within 12 hours of injury and before imaging prescription."

2. Similarly, please better clarify whether your usage in the Methods section of the "mTBI" inclusion population = mild TBI, or suspected mild TBI. These are different clinical entities.

Response: Thank you for this important comment. We agree that "mTBI" and "suspected mTBI" represent different clinical entities. To clarify this point, we have revised the Methods section to consistently use the term *clinically defined mTBI*. Patients will be enrolled only after assessment of predefined diagnostic criteria, including a plausible traumatic mechanism, compatible signs and/or symptoms of brain injury, and neurological evaluation consistent with mTBI (including GCS 13–15 assessed ≥30 minutes after injury). This terminology was adopted to make clear that inclusion is based on a predefined clinical definition of mTBI rather than on suspected mTBI alone. Several instances in which mTBI was used have been changed for clinically defined mTBI.

3. Please be more careful about using causal language in this non-randomized, observational study design (e.g., "its impact on clinically meaningful outcomes"). There are many other such examples.

Response: Thank you for this important comment. We agree that causal language should be avoided in the context of a non-randomized observational study. We have carefully revised the manuscript, particularly the Introduction and Objectives sections, to ensure that the wording consistently reflects the observational nature of the study design. Terms

implying causality have been replaced throughout by language more appropriate for an observational study.

Changes made in the manuscript introduction include:

- *Page 8 ¶ 2: "Blood biomarkers embedded in CDRs or as part of clinical guidelines can increase the objectivity of the assessment and the safety of managing mTBI in the ED" → "Blood biomarkers embedded in CDRs or as part of clinical guidelines may increase the objectivity of assessment and support the safe management of mTBI in the ED."*
- *Pagse 8 ¶ 2:"It has been established that…" → "Prior studies suggest that…"* when discussing management based on validated biomarker thresholds.
- *Page 9 ¶ 2:"its impact on clinically meaningful outcomes such as reducing CT utilization and shortening mTBI management time" → "its association with clinically meaningful outcomes, including CT utilization and mTBI management timelines."*
- *Page 9 ¶ 2: "assess the applicability and added value…" → "assess the feasibility, applicability and clinical performance…"*
- *Page 9 ¶ 2:"…to rule out the need for head CT scanning" → "…to support clinical decision-making regarding head CT use."*
- *Page 9 ¶ 2:"estimate the reduction in CT utilization achieved by implementing…" → "estimate changes in CT utilization associated with implementation…"*
- *Page 9 ¶ 2:"assess the impact of the proposed clinical pathway on these timelines" → "compare management timelines before and after implementation of the clinical pathway."*
- *Pagse 9 ¶ 2:"the economic effect of the new clinical pathway" → "the economic implications associated with the new clinical pathway."*

4. How do the authors propose to obtain a GCS from all patients "30 minutes after injury"? Can you provide preliminary data demonstrating that it is feasible to obtain a GCS that close to the time of injury? Are these pre-hospital GCS values?

Response:Thank you for this comment. This reflects an imprecise wording in the manuscript. The intended criterion was a Glasgow Coma Scale (GCS) score of 13–15 assessed 30 minutes or more after injury, rather than *30 minutes after injury*. Therefore, we are not requiring GCS assessment at a fixed time point close to the injury event, nor are we relying specifically on pre-hospital GCS measurements. The criterion is intended to reflect the standard mTBI definition, whereby GCS is assessed after the initial post-injury period.

Page 6 ¶ 2: Abstract: "mTBI will be defined using predefined clinical criteria including a

plausible traumatic mechanism, Glasgow Coma Scale score 13–15 assessed 30 minutes or more after injury, and compatible signs and/or symptoms of brain injury. Eligible patients must undergo blood sampling within 12 hours of injury and before imaging prescription."

Page 10 ¶3: Study population:" c) At least two acute symptoms related to the injury: feeling confused or disoriented, headache, nausea, vomiting, dizziness, vision problems, memory problems, emotional lability or irritability. d) GCS between 15 and 13, assessed 30 minutes or more after injury."

5. In the first paragraph of the "Intervention" section, it is not clear whether the pre-intervention study population overlaps with the mTBI definition provided in the "Study population" section. It appears to be different, which is a critical study design weakness.

Response: Thank you for this important comment. We agree that the original wording may have created ambiguity regarding the relationship between the study population and the pre-implementation cohort. Both the pre-implementation and post-implementation cohorts include the same clinically defined mTBI population, using identical inclusion and exclusion criteria as described in the *Study population* section. The difference between periods relates exclusively to the management strategy (standard care versus biomarker-guided clinical pathway), not to the definition of the enrolled population. We have revised the *Intervention* section to clarify this point explicitly.

Page 11 ¶1: "This study uses a before-and-after observational design to evaluate the implementation of a biomarker-guided clinical pathway for the management of clinically defined mTBI in the ED. In both the pre-implementation and post-implementation periods, patients will meet the same clinically defined mTBI inclusion and exclusion criteria described in the *Study population* section."

6. The authors use of past/present/future tenses in the Methods section raises confusion about which parts of the study have already been accomplished. Are you actively collecting data in the "post" time period? Was the "pre" phase actually done prospectively, or do the authors intend to just extract data from a historical population in their database?

Response:Thank you for this important comment. We agree that the use of mixed tenses may have created ambiguity regarding the study timeline and current study status. To clarify, both the pre-implementation and post-implementation phases involve prospective data collection. The pre-implementation phase began in July 2024 with prospective enrollment and collection of standard-of-care data; it is not based on retrospective extraction from a historical database. The VIDAS® TBI assay was

implemented in July 2025, and prospective enrollment and data collection are currently ongoing during the post-implementation phase. We have revised the *Study Setting* section to clarify the recruitment dates, implementation timeline, and the prospective nature of both study phases.

Study setting, page 10 ¶2: "The IMPACTS-BRAINI study is part of the BRAINI2 project, co-funded by the European Union through the European Institute of Innovation and Technology (EIT Health). This study is an investigator-initiated project conducted in collaboration with bioMérieux, which provided funding for the installation of a VIDAS® TBI analyzer and the associated biomarker tests. This prospective single-centre study will be conducted in the Emergency Department of Hospital 12 de Octubre, Madrid, Spain. Participant recruitment began in July 2024 with prospective enrollment and data collection during the pre-implementation phase. The VIDAS® TBI (GFAP, UCH-L1) assay was implemented in July 2025, after which prospective enrollment and data collection continued during the post-implementation phase. Completion of the post-implementation cohort is anticipated in July 2026."

7. In general, the Methods section does not provide sufficient detail to meet the standards for reproducible research. For example: "The effect of neurological comorbidities on test performance will also be assessed". There are many others.

Response: Thank you for this important comment. We agree that several aspects of the Methods section required greater detail to meet reproducibility standards. We have therefore revised different aspects of the methods section, included in different previous and later responses to the reviewer. We tried to clarify the different analysis that were planned as well as the definitions used for the different variables. We have changed the statistical considerations section in order to include clarifications on how the diagnostic performance, clinical effectiveness, safety analysis and subgroup analysis will be evaluated. We also expanded how neurological comorbidities will be identified and analysed.

Changes in the manuscript: Page 13 ¶1, 2 and 3: "The predictive performance of the predefined cut-off values for the two biomarkers will be validated in the entire study population calculating sensitivity, specificity, negative predictive value, positive predictive value and diagnostic accuracy for CT-detectable intracranial lesions. Receiver operating characteristic (ROC) curve analyses will also be performed, and the area under the ROC curve (AUC) of GFAP, UCH-L1, and the combined test, will be estimated as complementary measures of discriminatory performance. Patients in whom no head CT is performed will be classified as CT-negative. In addition, the clinical effectiveness of the biomarker-guided strategy will be evaluated by comparing CT utilization rates between the pre-implementation and post-implementation cohorts. Safety of the test will be evaluated through the occurrence of neurological deterioration, hospitalization, or subsequent Emergency Department visits within two weeks following mTBI.

The influence of age on diagnostic performance will be examined by stratifying patients into age groups (<65 and ≥65 years), with further subgroup analyses among patients aged ≥65 years (65–79 years and ≥80 years). Neurological comorbidities, including cognitive impairment, previous stroke, neurodegenerative disease, epilepsy, and chronic neurologic or psychiatric disorders documented in the medical record, will be identified. Subgroup and exploratory analyses will compare biomarker concentrations and diagnostic performance metrics of the predefined cutoffs between patients with and without neurological comorbidities.

To account for potential imbalances between the pre- and post-implementation groups with respect to demographic characteristics, comorbidities, treatments, or mTBI-related signs and symptoms, multivariable analyses will be performed as needed to adjust for confounding and to ensure comparability between groups. Secondary outcomes will likewise be analysed with stratification by age and comorbidity status."

8. How do the authors plan to capture "occurrence of neurological deterioration within two weeks following mTBI"? Will all enrolled patients, pre- and post-intervention, be actively contacted to determine whether or not one of these events has occurred? There is an EHR-check mentioned at "10-day post-TBI", but this does not align with the secondary safety outcome described earlier in the Methods section. **Response:**Thank you for this important comment. We agree that the wording in the manuscript was inconsistent. The reference to "10-day post-TBI" was an error and has been corrected to "two weeks after TBI" to align with the predefined secondary safety outcome.

Follow-up assessment will be performed for all enrolled patients in both the pre-implementation and post-implementation cohorts through review of routinely collected healthcare data; active patient contact will not be required. At two weeks after mTBI, clinical research associates will review all available information regarding patients' neurological status, need for hospitalization, and subsequent Emergency Department visits related to mTBI. This review will use information available through the institutional electronic health record as well as regional healthcare information systems in Madrid, including data from the local health authority and primary care/general practitioner records accessible through the HORUS platform. We have revised the Methods section to clarify the timing and sources of follow-up data collection.

Page 12 ¶4: "At two weeks after TBI, clinical research associates will review all available information regarding patients' neurological status, as well as any need for hospitalisation or subsequent Emergency Department visits related to mTBI. Follow-up data will be obtained through automatic extraction from the institutional electronic health record and review of regional healthcare records available through the Madrid health information systems, including the HORUS platform, which provides access to hospital and primary care records."

9. It appears from Figure 2 that the authors only plan to do the biomarker test on GCS 14 patients and some GCS 15 patients, but not on GCS 13 patients. Please better explain this decision-making, particularly in light of the robust evidence for using biomarkers to rule out the need for head CTs in suspected TBI patients with GCS 13-15.

Response:Thank you for this important comment. We agree that this decision-making process required clearer explanation. Although published evidence supports the use of biomarkers in patients with suspected TBI and GCS 13–15, our proposed pathway intentionally excludes patients with GCS=13 assessed ≥30 minutes after injury from biomarker-guided CT decision-making.

This decision is based on our previous experience with the combined GFAP/UCH-L1 test in the BRAINI multicentre observational study (Lagares A, de la Cruz J, Terrisse H *et al.* *eBioMedicine* 2024;110), in which a substantial proportion of patients with GCS=13 were recruited from our Emergency Department. In that cohort, approximately 50% of patients with GCS=13 assessed >30 minutes after injury had positive CT findings. In addition, unpublished observations from a larger series of patients managed in our ED show a similar CT positivity rate in this subgroup.

Given this high observed prevalence of intracranial lesions, together with the expected turnaround time of approximately one hour to obtain biomarker results through the core laboratory workflow, we consider that patients with GCS=13 assessed ≥30 minutes after injury should undergo early CT imaging without waiting for biomarker testing. We have revised the intervention section to better explain the rationale underlying this aspect of the clinical pathway.

Page 11¶2: "Following laboratory implementation of the VIDAS TBI assay, a revised clinical pathway incorporating serum GFAP and UCH-L1 testing was introduced as part of routine clinical care (Figure 2). Patients with GCS=13 assessed ≥30 minutes after injury are not managed using biomarker-guided CT decision-making because of the high observed prevalence of intracranial lesions in this subgroup in our previous and ongoing institutional experience, together with the expected turnaround time for biomarker results. "

Minor

1. As a small point of clarification, GFAP/UCH-L1 rule out *CT-positive* intracranial lesions. They do not rule out MRI-positive intracranial lesions. This is important to state clearly in your Abstract and throughout.

Response:
Thank you for this important clarification. We agree that the distinction between lesions

detectable by CT and those identifiable only by MRI should be stated more precisely. The intended use of the combined GFAP/UCH-L1 test in our study is to support identification of patients at low risk of CT-detectable intracranial lesions, in line with the current evidence base and intended clinical application of the assay. We have therefore revised the Abstract and manuscript throughout to explicitly refer to CT-detectable intracranial lesions where appropriate and to avoid broader wording that could incorrectly imply exclusion of MRI-detectable findings.

2. Please define CDR at its first usage

Response:

Done. Thanks.
